# Sleep quality and mental health during the COVID-19 pandemic in patients with severe obstructive sleep apnea

**DOI:** 10.1186/s41687-022-00454-x

**Published:** 2022-05-08

**Authors:** Lucia Spicuzza, Salvatore Mancuso, Raffaele Campisi, Carlo Vancheri

**Affiliations:** Dipartimento di Medicina Clinica e Sperimentale, UO Pneumologia, Azienda Policlinico-OVE, University of Catania, Via S. Sofia, 95123 Catania, Italy

**Keywords:** Obstructive sleep apnea, Sleep quality, COVID-19, Coronavirus Anxiety Scale, Depression, Anxiety, PSQI, CPAP

## Abstract

**Background:**

The first wave of the COVID-19 pandemic has produced remarkable effects on the sleep quality and mental status of the general population and more dramatic effects on patients with chronic illness. Patients with obstructive sleep apnea (OSA), already suffering from disordered sleep, might be more susceptible to the effect of the pandemic on their sleep quality and mental health. We therefore performed a case–control study to compare sleep quality, depression and anxiety symptoms reported by patients with severe OSA and age-matched healthy subjects during the first wave of the COVID-19. In June–July 2020 we enrolled a total of 222 patients with severe OSA, all treated with continuous positive airway pressure, and 164 healthy controls. Self-reported sleep quality was assessed using the Pittsburg Sleep Quality Index (PSQI). Symptoms of depression were assessed using the Patient Health Questionnaire module 9 (PHQ-9), while the specific “Coronavirus Anxiety Scale” (CAS) evaluated the level of anxiety.

**Results:**

Patients with OSA (61% males, 65 ± 9.6 years old, BMI 30.5 ± 3.6) and healthy controls had similar characteristics except for BMI slightly lower in controls. The perceived quality of sleep, referred to the pre-pandemic period, was significantly worse in patients with OSA than in controls. During the pandemic the rate of reported sleep disturbance increased from 54 to 66% in patients with OSA and from 29 to 40% in controls. A high percentage of patients and controls reported symptoms of depression (61% OSA and 65% controls), whereas lower levels of anxiety, similar in the two groups, were observed. In patients with OSA the PSQI score significantly positively correlated with the PHQ-9 score (r^2^ = 0.81) and the CAS score (r^2^ = 0.65).

**Conclusion:**

The rate of reported sleep disturbance in patients with OSA during the first wave of the COVID-19 pandemic is one of the highest evidenced in literature so far. As for the general population, in these patients there is a strict link between the perceived sleep quality and the psychological distress caused by the pandemic. A further deterioration of sleep quality is a fearsome event in the life of these patients who face life-long sleep problems.

## Introduction

Since its onset in December 2019, the ongoing COVID-19 pandemic has deeply affected worldwide people’s physical and mental health [1]. The risk to be infected, along with mobility restriction and the implementation of mitigation strategies, have been cause of psychological problems, such as depressive symptoms, anxiety and loneliness, extensively described among the general population across all continents [2]. A good sleep quality is essential when facing stressing situations, nevertheless one of the first complaints reported by the population, after the onset of the pandemic, has been insomnia and poor sleep quality affecting more than one third of the population across all involved countries with peaks over 50% recorded during the initial phase of the pandemic [3–6]. Sleep quality and psycho-somatic problems, including anxiety and depression, are strictly associated, in a bi-directional mode [7]. A poor sleep quality, resulting from a stressing situation, further worsens people’s state of anxiety, so that a vicious circle, difficult to break, is generated. The consequences of this vicious circle can be devastating. It has been shown that the risk of suicide has increased during the COVID-19 pandemic and insomnia has been proven to be a major cause of suicidal ideation [8]. In addition to the emotional stress, other risk factors for poor sleep quality include the individual susceptibility, the age and the presence of chronic somatic and psychiatric conditions [4, 9]. It is noteworthy that most of the studies available so far on the quality of sleep during the COVID-19 pandemic have been conducted through internet surveys among the general population, generally young adults, and among health care workers, whereas less is known about the elderly and those individuals suffering from chronic diseases.

Obstructive Sleep Apnea (OSA) is a chronic condition, acknowledged as major cause of sleep disturbance in the middle-aged population, in whom chronic sleep disruption is cause of psychological symptoms including anxiety and mild to severe depression. [10, 11]. Although treatment with continuous positive airway pressure (CPAP) abolishes apneic episodes and greatly improves psychological symptoms, still the emotional state and the quality of life of these patients remain suboptimal [10]. As the COVID-19 pandemic has produced remarkable effects on the mental status of the general population, its impact will likely be greater on individuals suffering from chronic illness. We therefore hypothesized that patients with OSA, who already suffer from disordered sleep, may experience a more dramatic change in sleep quality and mental health compared to healthy people during the pandemic. To test this hypothesis we performed a case–control study comparing perceived sleep quality, depression and anxiety symptoms in patients with severe OSA and in healthy subjects during the first wave of the COVID-19 pandemic. In order to assess these patient reported outcomes we used previously validated questionnaires including the Pittsburg Sleep Quality Index (PSQI), widely used to evaluate sleep quality, and the Patient Health Questionnaire module 9 (PHQ-9) for symptoms of depression. The “Coronavirus Anxiety Scale” (CAS) is a new tool recently introduced to measure the level of anxiety associated with the onset of the pandemic [12–17].

## Methods

### Study design, setting, sample size

In Italy, the first wave of the COVID-19 pandemic refers to a period between March and June 2020. This observational case–control study has been conducted in the Centre for Sleep Disorders, Respiratory Unit, University of Catania, between June and July 2020. During these 2 months we had a particularly high number of patients attending the Centre due to re-opening after the lock-down period. The study was carried out on the day on which patients with OSA using CPAP, all of them already followed in our centre, attended their scheduled follow-up visit. In fact, according to our local protocol, after the first year of CPAP treatment, patients attend a follow-up visit every year to undergo polygraphy, check compliance and for clinical evaluation. From a total of 231 patients attending the follow-up visit between June and July 2020 and considered eligible according to inclusion criteria, 5 patients refused to participate and 4 were unable to fill the questionnaires, so that 222 patients were finally included. We considered this sample to have sufficient power to detect differences between the groups, considering previous published data on the patient-reported outcomes that we were assessing [18, 19]. It has been calculated that to find a difference in the PSQI score between groups, 45 patients are sufficient in each group, based on an expected difference of 15% between groups with a standard deviation of 25% within groups [18]. For PHQ-9 it has been calculated that a sample size of 130 in two balanced groups will yield a power of at least 80% to detect standardized mean differences above 0.5 of a normally distributed variable [19].

### Participants

In this study we included patients with a diagnosis of severe OSA treated with nocturnal CPAP from at least 1 year. Inclusion criteria were: (1) apnea–hypopnea index (AHI) > 30 at diagnosis; (2) efficacy of CPAP assessed with a nocturnal polygraphy; (3) good compliance to the treatment assessed by records generated by the machine’s counter/software or by a remote monitoring software; (4) ability to fill the questionnaires and to sign informed consent. Exclusion criteria were: (1) patients with known psychiatric disorders or precedent symptoms of depression or previous use of any hypnotic or psychiatric drug; (2) patients in whom OSA was associated with any chronic neurological disorder; (3) patients with OSA uncontrolled by CPAP therapy. Healthy subjects, reporting no chronic illness, matched for age were used as controls. Controls were volunteers also recruited using advertisements and social networking sites. Data from controls were also collected, during June and July 2020.

### Intervention

Patient’s demographic and clinical records were available in our database since the diagnosis was made. In the study day clinical data were updated and the Epworth Sleepiness Scale (ESS) was performed to evaluate somnolence. The regular use of CPAP was assessed using records generated by the machine’s counter/software or by remote monitoring software. All data for the study were collected during one single visit. The patients were first invited to recall their sleep habits and quality before the pandemic onset and to fill a PSQI questionnaire (pre) as accurately as possible. Successively the patients were invited to fill a second PSQI questionnaire (post), focusing on their sleep quality in the months after the onset of the pandemic. Successively, two more questionnaires on symptoms of depression and anxiety experienced during the pandemic period were administered. A staff member was present to provide assistance if required. A single study day was also scheduled for controls. During this day demographic data and clinical history to exclude chronic illness were collected. The controls were asked to fill the same questionnaires filled by the patients, including the ESS, and an identical procedure was followed.

During the visit patients were informed that the questionnaires they were asked to fill were for research purpose and all signed an informed consent for collection of data and publishing. Also controls signed the same informed consent in the day they filled the questionnaires.

### Assessment of sleep quality

Sleep quality in patients and controls was evaluated using the PSQI [12]. This is a well validated and widely used self-rated questionnaire to evaluate overall sleep quality and disturbance. The questionnaire includes 19 items assessing seven aspects of sleep quality, including: subjective sleep quality, sleep efficiency, sleep latency, sleep duration, sleep disturbance, use of sleep medication, and daytime functioning. Each of these components provides a partial score and the total score, obtained by adding the score from each component, ranges from 0 to 21. A score greater than 5 indicates poor sleep quality and the greater it is, the worst is the quality. The PSQI has been used in its validated Italian version [13].

### Assessment of anxiety symptoms

The level on anxiety was assessed by the *“*Coronavirus Anxiety Scale” (CAS) a recently developed specific tool to assesses physiologically-based, dysfunctional anxiety symptoms associated with the fear of COVID-19 [14]. This five-item scale measures dizziness, sleep disturbance, tonic immobility, appetite loss, and abdominal distress on a five-point scale from 0 to 4. In a first validation study, the optimized cut-off score of ≥ 9 (90% sensitivity and 85% specificity) discriminated between persons with and without dysfunctional anxiety [14]. However, a replication study lowered the CAS cut-off score to ≥ 5 for the general public, as the sample in his original investigation comprised exclusively adults anxious about the coronavirus, whereas the sample in the replication study did not require experience related to COVID-19 [15]. The validated Italian version of CAS has been used for this study [16].

### Assessment of depression symptoms

To assess symptoms of depression we used the depression module of the Patient Health Questionnaire (PHQ), a well-known self-administered diagnostic instrument for common mental disorders. Specifically, the PHQ-9 is a module containing 9 items on a four-points scale (0–3). The test is commonly used as a screening tool for depression in primary care and it has been widely validated in the community. Cut-off point are: 5–9 mild depression, 10–14 moderate depression, ≥ 15 severe depression [17].

### Ethics

The study has been approved by the local ethical committee and signed consents to participate and to publish data were obtained for all participants.

### Statistical analysis

Descriptive statistic was used for demographic data presented as mean ± standard deviation (SD) or percentage for each sub-group. Differences among groups were analyzed using the appropriate t-test for unpaired or paired data. Categorical variables were analyzed using χ^2^ test. Pearson correlation test was used when appropriate. Multiple logistic regression analysis was used to determine independent predictors of poor sleep quality. P value was significant when < 0.05. Statistic was performed using SPSS software 14.0 (IBM, Armonk, NY USA).

## Results

We enrolled a total of 222 patients with OSA and 164 healthy controls. Demographic data of the study population are shown in Table 1. The groups were matched for age and only slightly differed for BMI, higher in patients with OSA. All patients had a severe OSA (AHI > 30) and were well controlled by the CPAP treatment in terms of AHI (mean value 4.1) and ESS (mean value 5.9), that was similar to healthy controls (mean value 4.9).

**Table 1:** Demographic and clinical data of the study groups

	OSA (n = 222)	Controls (n = 164)
Males, %	61	57
Age, years	65 ± 9.6	64.4 ± 8.7
BMI	30.5 ± 3.6*	27.3 ± 3.5
Smoking, %	18.6	19.1
Alcohol drinking, %	4.6	6.1
AHI basal	47 ± 16	
AHI during CPAP	4.1 ± 1	
Duration of CPAP treatment, years	7.3 ± 4.9	
ESS	5.9 ± 3.6	4.9 ± 2.3
CAS score	3.5 ± 2.1	3.8 ± 2.8
PHQ-9 score	8.3 ± 4.3	7.3 ± 4.1

OSA, obstructive sleep apnea; BMI, body mass index; CPAP, continuous positive airway pressure; AHI, apnea–hypopnea index; ESS, epworth sleepiness scale; CAS, Coronavirus Anxiety Scale; PHQ-9, Patient Health Questionnaire module 9

*P < 0.05 OSA versus controls

### Sleep variables and symptoms of depression and anxiety

During the first wave of the COVID-19 pandemic neither patients with OSA nor healthy subjects significantly changed their sleep habits. In the group with OSA 16% delayed bedtime and 21% anticipated by about 30 min, but most of them reported no change. In the control group only 9% anticipated while 15% delayed bedtime. The number of hours of sleep per night (6–7 h) was similar in the two groups and did not change during the pandemic, although sleep latency increased to a similar extent in both groups compared to the pre-pandemic period (Table 2).

**Table 2:** Changes in sleep variables and CPAP use before and during the first wave of the COVID-19 pandemic

	OSA	Controls	*P* value
*Sleep latency (minutes)*			
Before	21.7	17.9	0.3
During the pandemic	28.4*	26.9*	0.5
*Hours of sleep per night*			
Before	6.8 ± 1.2	7.1 ± 0.9	0.1
During the pandemic	6.7 ± 1.2	6.9 ± 0.9	0.2
*PSQI*			
Before	6.4 ± 2.0	4.6 ± 2.0	< 0.001
During the pandemic	7.5 ± 2.3*	6.0 ± 2.5*	< 0.001
*CPAP use per night (hours)*			
Before	6.7 ± 1.6		
During the pandemic	6.8 ± 1.2		

OSA, obstructive sleep apnea; PSQI, Pittsburg Sleep Quality Index; CPAP, continuous positive airway pressure

*P < 0.001 before versus during the pandemic

**Fig. 1:**
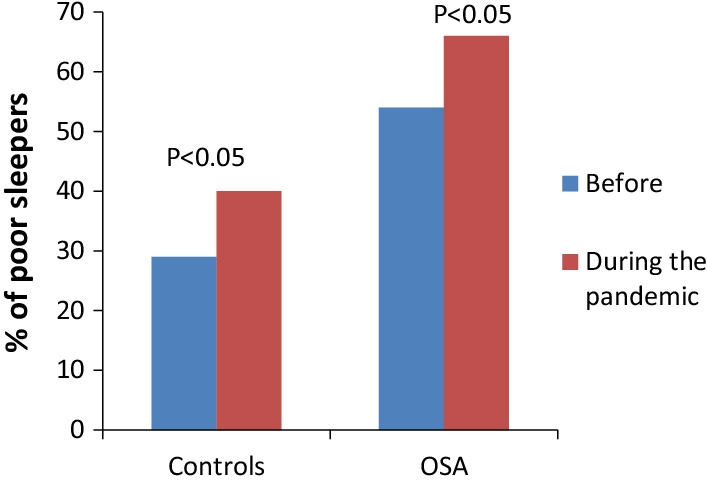
Prevalence rate of poor sleepers (PSQI > 5) among OSA and controls before and during the first wave of the COVID-19 pandemic

The mean PSQI score, in the pre-pandemic period was significantly higher in patients with OSA than in controls (6.4 ± 2.0 vs 4.6 ± 2.0, *P* < 0.001). In both groups the mean PSQI score significantly increased during the pandemic (7.5 ± 2.3 OSA and 6.0 ± 2.5 controls, *P* < 0.001) (Table 2). In the group with OSA the percentage of patients with a PSQI > 5, the cut-off value for defining poor sleep quality, was 54% before and 66% during the pandemic (*P* < 0.05), whereas in the control group the rate of poor sleepers increased from 29 to 40% (*P* < 0.05) (Fig. 1).

Results obtained from the CAS questionnaire, evaluating COVID-19-related anxiety symptoms, revealed a similar level of anxiety in the two groups (mean scores shown in Table 1). Twenty-eight percent of patients with OSA and 24% of the controls (*P* = 0.6) reported an abnormal level of anxiety (Table 3).

**Table 3:** Subgroups data for PSQI, CAS and PHQ-9 score during the first wave of the COVID-19 pandemic

	OSA (n = 222)	Controls (n = 164)	*P* value
*PSQI*			
6–9	107(48%)	46 (28%)	< 0.001
> 10	40 (10%)	20 (12%)	0.1
*CAS score*			
5–9	42 (19%)	32 (19%)	0.8
> 9	22 (9.9%)	8 (4.8%)	0.4
*PHQ-9 score*			
5–9	92 (44%)	60 (36%)	0.4
> 9	44 (19%)	48 (29%)	< 0.05

OSA, obstructive sleep apnea; PSQI, Pittsburg Sleep Quality Index; CAS, Coronavirus Anxiety Scale; PHQ-9, Patient Health Questionnaire module 9

A high percentage of patients and controls showed symptoms of depression, measured trough the PHQ-9 questionnaire (61% OSA and 65% controls). In most of the cases symptoms of mild depression were complained, however a higher number of healthy subjects reported symptoms of moderate to severe depression compared to patients (29% vs 19% respectively, *P* < 0.05) (Table 3).

### Factors influencing the quality of sleep in patients with OSA

**Fig. 2:**
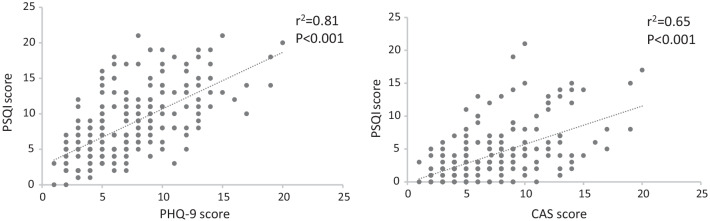
Correlation between PSQI score, CAS and PHQ-9 score in patients with OSA during the pandemic

In order to understand factors affecting the quality of sleep in the group of patients with OSA we compared patients with PSQI > 5 and those with PSQI < 5 and found no difference in the mean age, AHI, BMI and gender. The mean ESS was significantly higher in patients with PSQI > 5 compared to those with PSQI < 5 (6.3 ± 2.6 vs 4.9 ± 1.9, respectively, *P* < 0.05). Linear regression analysis showed that the PSQI score significantly positively correlated with the CAS score (r^2^ = 0.65, *P* < 0.001) and the PHQ-9 score (r^2^ = 0.81, *P* < 0.001) (Fig. 2). To assess independent predictors of poor quality of sleep a multiple logistic regression analysis was performed using the PSQI score as the dependent variable and age, BMI, CAS score, PH-9 score and ESS as the independent variables. We found that the PH-9 score was the strongest predictor of abnormal PSQI score followed by CAS score and ESS (Table 4).

**Table 4:** Multiple logistic regression to assess predictors of PSQI score in patients with OSA

	Odd ratio	95% Confidence interval	*P* value
Age	1.01	0.96–1.06	0.69
BMI	1.03	0.9–1.23	0.42
PHQ-9 score	1.27	1.05–1.45	< 0.001
CAS score	1.12	0.91–1.37	< 0.05
ESS	1.19	1.05–1.45	< 0.05

PSQI, Pittsburg Sleep Quality Index; OSA, obstructive sleep apnea; BMI, body mass index; PHQ-9, Patient Health Questionnaire module 9; CAS, Coronavirus Anxiety Scale; ESS, Epworth Sleepiness Scale

## Discussion

The results of this study clearly indicate that patients with severe OSA, although well controlled by CPAP treatment, have a poor perceived sleep quality compared to healthy controls that has greatly worsened after the onset of the COVID-19 pandemic. Although high rates of sleep disturbance have been reported across all countries during this dreadful event, it is noteworthy that the prevalence rate of sleep disturbance that we found among patients with OSA (66%) is exceptionally high. In these patients we showed a strict correlation between sleep disturbance and the level of anxiety and depression caused by the pandemic. The prevalence of these symptoms did not differ between OSA and healthy subjects.

To our knowledge this is the first study investigating the effect of the COVID-19 pandemic on perceived sleep quality and psychological stress in patients with obstructive sleep apnea. Anxiety, depressive mood and sleep disturbance have been broadly reported during lockdown periods following the first wave of the COVID-19 pandemic, from March to June 2020 [3, 5, 6]. The emotional distress has been associated with the fear to be infected, forced home confinement and social isolation, economical instability and worries about the future [4]. During this period approximately one third of previously good sleepers complained the occurrence of sleep disturbance across all continents [2, 3].

Italy, where this study was carried out, has been the gateway of the pandemic to Europe in February 2021. For this reason, in this country, one of the highest level of emotional distress in the world has been recorded. Casagrande et al. in an early study showed that, after the COVID-19 outbreak, among 2291 subjects from the general population, 57.1% complained poor sleep quality. A significant relationship was evidenced between sleep quality and generalized anxiety or symptoms of post-traumatic distress [6]. These data were comparable to those reported in China after the outspread in Wuhan in December 2019 [5].

One first observation is that during lockdown periods home confinement has produced a change in people’s sleep habits as generally the population confined at home delayed bed-time and slept longer than usual [20]. However, it is noteworthy that a longer time spent in bed simply reflects more time spent at home and does not indicate a good quality of sleep, that in fact worsens [21]. In our sample, most of the patients and healthy controls did not change their bed-time and total time spent in bed in the pandemic period. Nevertheless, in line with earlier observations, sleep latency significantly increased in both [22]. It is noteworthy that during the pandemic our patients showed an optimal adherence to CPAP treatment (mean average use > 6 h/night), that, differently from what it has been recently reported in a large cohort study, was not different from the pre-pandemic period [23].

An altered sleep pattern is an hallmark of untreated OSA and up to 100% of the patients report a subjective sensation of poor sleep quality with a PSQI score well above the cut-off limit [24]. Treatment with CPAP clearly improves sleep architecture, restoring sleep stages and reducing the number of arousals [25]. Though, the subjective sensation of satisfying sleep can be differently affected, perhaps to the discomfort given by the CPAP itself. Our patients, well controlled and adherent to CPAP treatment, had an high score at the PSQI questionnaire referring to the pre-pandemic period, with 54% of the patients reaching the score of a poor sleeper compared to 29% of the controls. This is not surprising, as a previous study on patients treated with CPAP reported a mean PSQI score of 6.6, in good agreement with our mean score of 6.4 [18]. Although during the pandemic the rate of self-reported sleep disturbance increased to a similar extent in patients and in healthy controls, the rate in patients with OSA was exceptionally high. It is conceivable therefore that the increased difficulty in sleeping and the COVID-19-related emotional distress may further contribute to worsen health quality and quality of life in these patients.

This is the first study addressing sleep issues during the pandemic in OSA, however few previous studies have been carried out in patients affected by other chronic conditions [26–28]. One large study in UK showed that, during the pandemic, older people with physical disabilities had more symptoms of depression and anxiety compared to people without a physical disability. Self-reported poor sleep quality was evidenced in 49.9% of people with disability and in 39.5% of people without [26]. These differences were accompanied by lower levels of social contact in people with disability, while patients with OSA, in most of the cases live with their family, regularly work and have a normal social life.

Another recent study evaluated the impact of the COVID-19 pandemic on patients with at least one chronic non-communicable disease including hypertension, diabetes, cardiovascular or respiratory disease. Compared to healthy controls, people with such chronic conditions had a 36% greater chance of impaired sleep, strictly associated with a state of emotional stress [27]. The high level of psychological distress in patients with chronic diseases has been explained by the anxiety arising from the fear of contagion, as this event may be particularly dangerous in this fragile population [27]. It is now acquired that patients with OSA are at high risk of developing severe COVID-19 [29]. Our data indicate that although a number of patients complained symptoms of anxiety and depression, still the proportion was similar to the control group, indicating that the emotional distress in these patients is not divergent from the general population. Indeed the rate of self-reported sleep disturbance that we have found in patients with OSA is the highest, compared to previous data reported in the literature in the general population and in other chronic conditions [4]. This high prevalence is explained by the fact that in basal condition the perceived quality of sleep in these patients is poor and in the pandemic period it is worsened by worries that affect also healthy subjects. In fact, we found a strict correlation between the PSQI and the PHQ-9 and, to a lesser extent, the CAS score. Thus, symptoms of depression are a strong determinants of poor sleep quality. Depression is common in patients with OSA, although, to avoid confusion, we excluded from the study those who reported psychological symptoms before initiating CPAP. Of course we cannot completely exclude the presence of previous depression/anxiety, however this is unlikely as our patients scored similarly to healthy subjects. Sleep disturbance reduces daytime functioning further worsening the general state of anxiety/depression, so that a vicious circle, difficult to break, is generated. A remarkable study has shown that the level of anxiety about COVID-19 positively correlated with insomnia severity and suicidal ideation [8]. Clearly, the high prevalence of sleep disturbance may be devastating in fragile individuals as patients with OSA are. On limitation of this study is that we used a PSQI questionnaire not only to assess current sleep quality, but also to collect data referring to the a previous period, before the on-set of the pandemic, so we assumed that some details could be omitted or forgotten. Although this use of the PSQI is unusual, we felt that patients were quite confident when recalling their sleep habits before the pandemic. In addition, the same approach was used for controls and data obtained in our controls were perfectly in line with previous published data.

In conclusion, our data indicate that in patients with severe OSA treated with CPAP the quality of sleep, already poor before the pandemic, dramatically worsened after the onset of the COVID-19 pandemic. The prevalence rate of perceived sleep disturbance that we found in OSA is the highest reported in the literature, among healthy individuals or patients affected by other chronic diseases. During the first wave of the pandemic the level of emotional stress in patients with OSA did not differ from healthy subjects and was strictly associated with the occurrence of sleep disturbance. A further deterioration of sleep quality is a fearsome event in the life of patients who face life-long sleep problems, therefore great attention must be paid to their mental health.

## Data Availability

The datasets used and/or analysed during the current study are available from the corresponding author on reasonable request.

## Abbreviations

AHI: Apnea-hypopnea index
CAS: Coronavirus Anxiety Scale
CPAP: Continuous positive airway pressure
ESS: Epworth Sleepiness Scale
OSA: Obstructive sleep apnea
PSQI: Pittsburg Sleep Quality Index
PHQ-9: Patient Health Questionnaire
